# Nitric oxide as a mediator of inflammation?—You had better believe it

**DOI:** 10.1155/S0962935195000639

**Published:** 1995-11

**Authors:** Mark J. S. Miller, Matthew B. Grisham

**Affiliations:** 1 Department of Pediatrics Louisiana State University Medical Center New Orleans LA USA; 2 Department of Physiology & Biophysics Louisiana State University Medical Center Shreveport LA USA

## Abstract

Nitric oxide has enigmatic qualities in inflammation. In order to appreciate the precise contributions of nitric oxide to a pathophysiological process, one must account for enzyme source, coproduction of oxidants and antioxidant defences, time, rate of nitric oxide production, cellular source, peroxynitrite formation and effects on DNA (mutagenesis/apoptosis). We contend that there is ample evidence to consider nitric oxide as a molecular aggressor in inflammation, particularly chronic inflammation. Therapeutic benefit can be achieved by inhibition of inducible nitric oxide synthase and not the donation of additional nitric oxide. Furthermore, there is growing appreciation that nitric oxide and products derived thereof, are critical components linking the increased incidence of cancer in states of chronic inflammation.


Mediators of Inflammation 4, 387–396 (1995)

### Nitric oxide as a mediator of inflammation?—You had better believe it

Mark J. S. Miller[1,CA] and Matthew B. Grisham[2]

[1]Department of Pediatrics, Louisiana State University Medical Center, New Orleans, LA, USA; and [2]Department of Physiology & Biophysics, Louisiana State University Medical Center, Shreveport, LA, USA

[CA]Corresponding Author

**NITRIC oxide has enigmatic qualities in inflammation. In order to appreciate the precise contributions of nitric oxide to a pathophysiological process, one must account for enzyme source, co-production of oxidants and antioxidant defences, time, rate of nitric oxide production, cellular source, peroxynitrite formation and effects on DNA (mutagenesis/apoptosis). We contend that there is ample evidence to consider nitric oxide as a molecular aggressor in inflammation, particularly chronic inflammation. Therapeutic benefit can be achieved by inhibition of inducible nitric oxide synthase and not the donation of additional nitric oxide. Furthermore, there is growing appreciation that nitric oxide and products derived thereof, are critical components linking the increased incidence of cancer in states of chronic inflammation.**

**Key words:** Apoptosis, inflammation, immune, mutagenesis, peroxynitrite

### Solution chemistry of nitric oxide

Organic chemists have known for some time that cigarette smoke and atmospheric pollutants generated from the combustion of petroleum-based and other organic products (automobile exhaust, industrial smoke) contain high concentrations of a variety of oxidized metabolites of nitrogen (Table 1). Many of these same nitrogen oxides, collectively called $NO_x$ and which are ultimately derived from NO, are produced by mammalian cells.

NO is a colourless gas that contains an odd number of electrons and is, by definition, a free radical. The unpaired electron is delocalized over the nitrogen and oxygen atoms. NO is relatively unstable in the presence of molecular oxygen with an apparent half-life of approximately 3–5 s[1]. It will rapidly decompose in the presence of $O_2$ to yield a variety of nitrogen oxides in a complex series of interactions:[1,2]

$$2NO + O_2 \longrightarrow 2NO_2$$

$$2NO + 2NO_2 \longrightarrow 2N_2O_3$$

$$2N_2O_3 + 2H_2O \longrightarrow 4NO_2^- + 4H^+$$

where $NO_2$, $N_2O_3$ and $NO_2^-$ represent nitrogen dioxide, dinitrogen trioxide and nitrite, respectively.

Although this reaction pathway has been proposed to occur in aqueous (i.e. physiological) solutions, there is some controversy as to whether $N_2O_3$ is formed as an intermediate.[3,4] There is no doubt that the only stable product formed by the spontaneous auto-oxidation of NO in oxygenated solutions is $NO_2^-$.[2] However, when urine or plasma are analysed, predominantly $NO_3^-$ is found in these extracellular fluids. Although the mechanisms by which NO is converted to $NO_3^-$ *in vivo* are not entirely clear, there are at least two possibilities. Ignarro *et al.*[2] have suggested one mechanism in which the $NO_2^-$ derived from NO autooxidation is rapidly converted to $NO_3^-$ via its oxidation by certain oxyhaemoproteins ($P\text{–}Fe^{2+}O_2$) such as oxyhaemoglobin or oxymyoglobin:

$$2P\text{–}Fe^{2+}O_2 + 3NO_2^- + 3NO_2^- + 2H^+ \longrightarrow 2P\text{–}Fe^{3+} + 3NO_3^- + H_2O$$

or

$$4P\text{–}Fe^{2+}O_2 + 4NO_2^- + 4H+ + 4H^+ \longrightarrow 4P\text{–}Fe^{3+} + 4NO_3^- + O_2 + 2H_2O$$

It should be noted, however, that these investigators used large concentrations of NO (300 μM) which will rapidly auto-oxidize to $NO_2^-$. Although the authors suggested that the $NO_2^-$ would in

**Table 1.** Oxidized metabolites of nitrogen

| Symbol | Name | Comments |
|---|---|---|
| $NO^•$ | Nitric oxide | Free radical |
| $NO_2^•$ | Nitrogen dioxide | Free radical; oxidizing agent |
| $N_2O$ | Nitrous oxide | Anaesthetic |
| $N_2O_3$ | Dinitrogen trioxide | Nitrosating agent |
| $N_2O_4$ | Dinitrogen tetroxide | Dimetric $NO_2^•$; nitrosating agent |
| $NO_2^-$ | Nitrite | Produces nitrosating agent at acidic pH |
| $NO_3^-$ | Nitrate | Stable anion |

© 1995 Rapid Science Publishers

**Table 2.** Factors influencing the role of nitric oxide in inflammation

1. Oxidant levels and metal-binding sites
2. Enzyme source (cNOS only *vs.* iNOS)
3. Time (iNOS expression, repair *vs.* injury)
4. Concentration (products of second-order kinetics: $N_2O_3$, $N_2O_4$)
5. Cellular source
6. Interactions with other oxidants/substrates ($ONOO^-$)
7. Gene expression/DNA damage (mutagenesis, apoptosis)

turn react with the haemoproteins, this reaction is quite slow, requiring 2–3 h.[2] A second, possibly more reasonable explanation for the presence of predominantly $NO_3^-$ *in vivo* may have to do with the fact that the levels of NO produced by nitric oxide synthase (NOS) *in vivo* would be much smaller and thus the half-life of NO would be much longer. In this case, NO would react directly and very rapidly with oxyhaemoproteins to yield $NO_3^-$ before it has an opportunity to auto-oxidize to $NO_2^-$.[5]

Thus, it is within this background that the complexity of NO chemistry can be appreciated. A family of oxidative products is generated under physiological conditions. These oxidative products may have similar or contrasting effects to the parent nitric oxide or alternatively no biological consequence (Table 2).

### Mechanisms of NO-dependent tissue injury and dysfunction

*Metabolic inhibition:* Several groups of investigators have demonstrated that the large amounts of NO produced from activated macrophages are capable of injuring hepatocytes, pancreatic islet cells, and lymphocytes. The mechanisms by which NO injures cells appears to include intracellular iron release, inhibition of mitochondrial function and inhibition of DNA synthesis. It is known for example, that NO will inhibit three mitochondrial enzymes including acotinase (tricarboxylic acid cycle), NADPH–ubiquinone oxidoreductase and succinate–ubiquinone oxidoreductase (Complex I and Complex II or the mitochondrial respiratory chain).[6–8] This inhibition is a result of the NO-mediated degradation of the iron–sulfur clusters associated with these three enzymes. This interaction most probably accounts for the NO-mediated release of nonhaem iron from certain target cells.[9] In addition, NO has been shown to inactivate ribonucleotide reductase, which may account for its ability to inhibit DNA synthesis.

*Influence of time:* There is a wealth of literature indicating either that NO donors protect or NOS inhibitors exacerbate tissue injury to an acute insult. Virtually all these studies have all been acute in nature, i.e. experimental protocols were only a few hours in duration. One may pose the question, "How can time influence the role of NO in a state of inflammation?" The answer lies primarily in expression of iNOS. Nitric oxide promotes tissue injury when it is produced in excess, but the isoform which produces large quantities of NO (iNOS) is regulated at the transcriptional level. In other words, to proceed from stimulus and activation of iNOS expression via transcription of the gene and its translation into new iNOS protein and the formation of excess NO, takes a defined period of time. Under ideal circumstances this process takes several hours but in a disease where the stimuli may vary in amount and relative importance, then the expression of iNOS may be delayed even further. Until this point is reached only limited production of NO can be achieved.

An excellent example of this phenomenon has come from the laboratory of Drs Brendan Whittle and Salvador Moncada. In 1990 they reported that the NO donor *S*-nitroso-*N*-acetylpenicillamine attenuated endotoxin-induced acute intestinal damage,[10] suggesting that NO was protective over the 3 h of the experimental protocol. In a follow-up study reported 4 years later,[11] they confirmed the initial report; the NOS inhibitors L-NAME or L-NMMA, exacerbated endotoxin-induced vascular injury when administered at the same time as endotoxin and evaluated 2 h later. If, however, administration of the NOS inhibitors was delayed until 3 h after endotoxin administration and the protocol extended for an additional 2 h (total of 5 h) then the NOS inhibitors were protective. Thus, in the matter of hours NO had transformed from an agent which was anti-inflammatory to one which was now pro-inflammatory. Although not confirmed by this group, the likely reason for this transformation is the time required for the expression of iNOS. Endotoxin is a classic stimulant for the expression of iNOS and expression of iNOS has been noted after ischaemia/reperfusion injury.[12]

Few studies have addressed the effects of NO donors on a chronic inflammatory process, although there is ample evidence of NOS inhibitors being therapeutic in chronic inflammation.[13–16] Our colleagues in this debate have shown in models of gastric ulceration that NSAIDs that contain a NO-donating moiety are protective when compared to the parent compound without the NO donor structure.[17,18] The mechanisms for this phenomenon are not clear. While a vascular effect and a role of cGMP-based mechanisms has been proposed, an effect on microbial flora remains a possibility. Nitric oxide is a mediator of host defence and any limitation

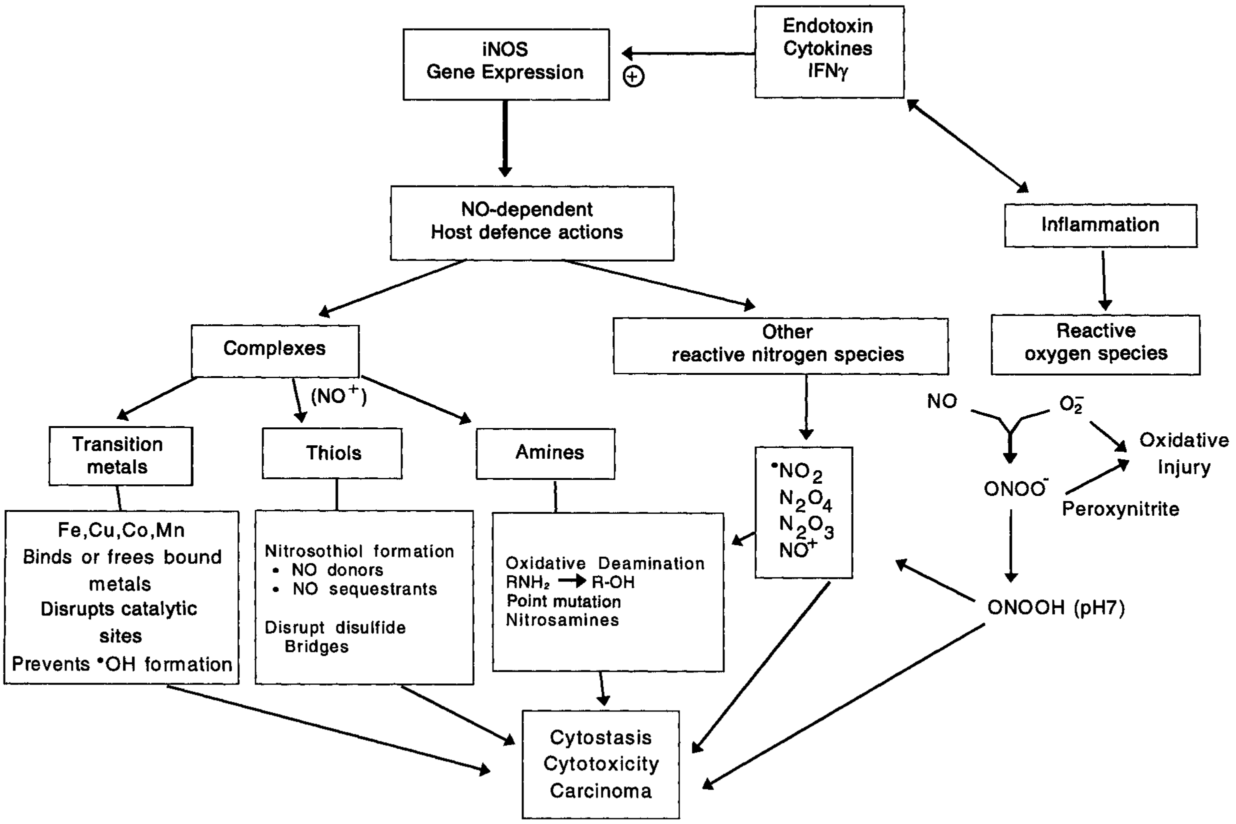

FIG. 1. Sites of action and interaction by nitric oxide in inflammation.

on bacterial translocation at the site of the ulcer may potentially promote healing.

Are there conditions where cNOS acts like iNOS; and if so, under these conditions should NOS inhibition be protective even if the protocols were acute? The answers to these questions are yes and yes! Stroke is an acute condition associated with an immediate and sustained release of NO, and neuronal injury can be ameliorated by attenuating the activity of the neuronal isoform of cNOS (nNOS). Neuronal NOS, like the endothelial constitutive isoform, eNOS, is regulated by the intracellular levels of calcium and activation of calmodulin. In contrast, the inducible isoform, iNOS, has calcium-activated calmodulin as an integral subunit, and therefore iNOS activity is independent of intracellular calcium fluxes.[19] In stroke NMDA receptor activation maintains intracellular calcium at a high level. Consequently nNOS activation is sustained, as opposed to the normal pulsitile manner, and as a result, large, sustained release of NO results in exacerbation of the initial neuronal injury. There are reports that NOS inhibitors are not neuroprotective.[20,21] This can be reconciled as the result of impairing endothelial NOS activity and exacerbating the vascular complications and granulocyte infiltration response associated with stroke. These contrasting roles of the neuronal and endothelial NOS isoforms in stroke have been highlighted by the application of stroke models to nNOS knock-out mice.[22] In animals lacking nNOS the administration of an NOS inhibitor resulted in an exacerbation of the injury, when studied acutely. Furthermore, there is evidence that the redox state of NO may account for[23] neuroprotective or neurodestructive roles.

If nNOS acts like iNOS in acute stroke is there a role for iNOS itself in stroke? It appears that if the protocol length is extended an iNOS-only contribution to the overall condition[24] can be seen. Inducible NOS can be expressed in neurons and glia.[25–27] In this respect there is an inflammatory component to cell death in stroke, this component involves iNOS and is apparent hours to days after the initiation of neuronal injury. Administration of aminoguanidine, an NOS inhibitor with selectivity for iNOS, was beneficial in experimental stroke when administered 6 h after the initiation of stroke.[28]

*Influence of enzyme source—iNOS* vs. *cNOS:* With the exception of acute neuronal injury associated with stroke, a reasonable generalization is that one can predict the role nitric oxide will play in a disease process by determining the presence or absence of iNOS. If iNOS is absent then NO will primarily exhibit anti-inflammatory effects. Conversely, the presence of iNOS is an excellent indicator that NO is directly contributing to tissue injury. This generalization is blurred in states of infection where NO generated from iNOS is necessary for killing the invading microorganisms and inhibition of NO formation may promote the growth and extent of infiltration of this infection and enhance the resultant tissue injury. Nevertheless, there is evidence that in experimental pneumococcal meningitis excess NO formation contributes to the brain oedema and meningeal inflammation.[29] For the purposes of this debate we will exclude the complications of infection, focusing rather on autoimmune diseases or non-infectious inflammation.

There are relatively few studies which have directly assessed iNOS gene expression in disease states, particularly over the time-course of the inflammatory condition. In models of gut inflammation we have observed iNOS gene expression at the onset of symptoms in the TNBS (trinitrobenzene sulfonic acid) model of ileitis and colitis[30] and adjuvant ileitis. In the TNBS model iNOS gene expression is apparent 1 day after the administration of TNBS and maintained for the first week of inflammation, as determined by RT-PCR and Western blotting. In contrast, in the adjuvant model of ileitis, in an analogous manner to adjuvant arthritis, the onset of symptoms is delayed until about 10 days after administration of the adjuvant. In keeping with the role of iNOS in the initiation of inflammation, the expression of iNOS is apparent in this model at day 14 but not at day 7. Similarly, increased production in HLA-B27 transgenic rats is only noted at the onset of symptoms and persists for the duration of the spontaneous colitis that they exhibit.[31]

The expression of iNOS has also been reported in models of arthritis initiated by adjuvant[16] and streptococcal cell wall fragments.[14] The expression of iNOS coincides with increased production of nitric oxide and the initiation of inflammatory symptoms. While the loss of matrix proteoglycans from articular cartilage is a hallmark of destructive joint disease like osteoarthritis or rheumatoid arthritis and may well involve NO-related mechanisms, it is now appreciated that nitric oxide can inhibit glycosaminoglycan synthesis.[32–34] In other words natural repair mechanisms may also be compromised by nitric oxide. While this is compelling circumstantial evidence suggesting that NO is involved in the disease process, these associations do not reveal the role of NO in that process. Nevertheless, with chronic administration of NOS inhibitors it is readily demonstrated that the expression of iNOS and increased synthesis of NO in inflammation is a major contributor to tissue injury in these states of inflammation.

Initially, therapeutic benefit was demonstrated by pharmacological probes which were not selective for iNOS (either L-NAME or L-NMMA) in TNBS ileitis[13] streptococcal cell wall arthritis or colitis[14,15] or adjuvant arthritis,[32,33,35] or spontaneous glomerulonephritis in mice.[36] With the availability of inhibitors with greater selectivity for inducible NOS, e.g. aminoguanidine or L-NIL (*N*-iminoethyl-L-lysine) therapeutic benefit has also been observed in a range of chronic inflammatory states, e.g. peptidoglycan–polysaccharide induced chronic granulomatous colitis,[15] adjuvant arthritis,[16] TNBS and adjuvant ileitis[26,30] and autoimmune encephalomyelitis in SJL mice.[37]

We have also described a mild ileitis induced by chronic administration of the NOS inhibitor, L-NAME, in otherwise normal guinea-pigs.[38,39] While superficially this is consistent with a loss of the physiological anti-inflammatory actions of nitric oxide, when we further addressed indices of nitric oxide formation there was no evidence of attenuated NO formation. Rather, all indices suggested that nitric oxide formation was increased in conjunction with increased levels of cGMP. In order to explain this perplexing phenomenon we addressed iNOS gene expression and noted that chronic administration of L-NAME results in the expression of iNOS. This response was not isolated to the gut, being apparent in lung, vasculature and uterus, in rats and guinea-pigs. It appears that chronic reductions in NO release can result in the expression of iNOS in order to compensate for inadequate levels of NO. This effective mechanism for restoring NO production is not without complications as iNOS, once formed, is poorly regulated and can result in excess NO formation which may account for the leukocytosis, ileitis and increased endothelin levels associated with chronic L-NAME administration.[38,40] This phenomenon is probably only achievable because L-NAME is slightly selective for cNOS; iNOS selective inhibitors do not display this response. Thus it is important to verify if the administration of an NOS inhibitor has reduced NO formation, particularly if administered chronically, otherwise the data may be interpreted incorrectly.

If NO is primarily anti-inflammatory, states of tissue injury and inflammation should be characterized by a reduction in NO levels. This is not the case; states of inflammation are virtually always characterized by increased NO levels. This is more consistent with the pro-inflammatory role of NO, otherwise the interpretation is that NO is anti-inflammatory but its efficacy is quite weak.

As many of the disease states demonstrating a pro-inflammatory role of NO via expression of iNOS are autoimmune in character, it is logical to expect that organ transplant rejection responses may also involve iNOS. Recently Worrall *et al.* described that iNOS is expressed in allogenic cardiac transplants in rats during the rejection response, in association with increased systemic levels of nitrate/nitrite.[41] Further, administration of the iNOS inhibitor, aminoguanidine, prolonged graft survival and significantly reduced histological indices of rejection.

There is an expanding list of induced and spontaneous disease states which are characterized by exaggerated release of nitric oxide and inhibitors of NOS display therapeutic efficacy. These include HLA B27 transgenic rats with spontaneous intestinal inflammation,[31] colonic inflammation induced by the sulfhydryl blocker, iodoacetamide,[42] insulin-dependent diabetes mellitus,[43] carrageenan-induced pleurisy and footpad oedema,[44,45] endotoxin-induced uveitis,[46] air pouch models of granuloma formation,[47] TNBS and acetic acid colitis in rats[48,49] and spontaneous, idiopathic colitis in rhesus macaques.[50]

Nitric oxide displays anti-inflammatory characteristics in ischaemia-reperfusion injury.[51–53] These protocols are acute and therefore are iNOS-independent models, although with extension of the study period iNOS can be detected in previously injured tissue.[12] Recently, a report has addressed the role of nitric oxide in ischaemia-reperfusion injury in a condition where iNOS is present, i.e. after administration of endotoxin. Ma and colleagues noted that reperfusion injury was attenuated by the NOS inhibitor L-NMMA under these conditions, as opposed to the usual exacerbation seen in the absence of endotoxin.[54] They proposed, based on the evaluation of chemiluminescence signals, that excess NO reacted with superoxide induced by ischaemia-reperfusion, to form the highly toxic peroxynitrite, which in turn contributed to hepatocellular injury. This is another example of NO promoting tissue injury when conditions are favourable, viz. during states in which iNOS is expressed.

In human disease there remains a lack of solid evidence that inhibition of NOS is protective, largely because compounds with sufficient selectivity for iNOS are not available for testing. The exception is the preliminary work in septic shock with L-NMMA.[55] In shock a complete withdrawal of nitric oxide synthesis may be deleterious, leading to suggestions that scavengers that mop-up excess NO may be viable alternatives. One interesting NO scavenger is hydroxocobalamin, a form of vitamin B12 lacking cyanide, which prevents and reverses endotoxic shock in rats and mice.[56]

Nevertheless, there is ample evidence of excess nitric oxide formation in human diseases, including ulcerative colitis,[57–59] gastritis[60,61] and arthritis.[62] Biochemical or immunohistochemical evidence suggests that iNOS is expressed in these disease states and probably accounts for the increased production of nitric oxide. We postulate that the role of this exaggerated production of nitric oxide in these human diseases will parallel animal models, i.e. nitric oxide will be pro-inflammatory.

*Cellular sources of iNOS in inflammation:* Traditionally, iNOS is associated with macrophages and to a lesser extent granulocytes, because these are the cell types in which iNOS activity was discovered. It is now apparent that many cell types can express iNOS but in contrast, the neuronal and endothelial forms of NOS remain localized in their original classification with the exception of epithelial cells and skeletal muscle which express cNOS.[63] Thus, in an inflammatory disease state iNOS is expressed in a variety of cells and not just in infiltrating leukocytes. In arthritis, chondocytes, osteoblasts and synovium can be a source of NO.[16,32,64,65] In gut inflammation the predominant sources of iNOS are epithelia[26,30,66,67] and the enteric nervous system,[26,30] in addition to resident and infiltrating leukocytes in the lamina propria. Tepperman and colleagues first described the ability of endotoxin to increase NO synthesis in cultured epithelial cells, a response which led to epithelial cell death via NO-dependent mechanisms.[66,67] Furthermore, it appears that the epithelium responds to this $NO_x$-induced stress and resultant DNA damage by undergoing apoptosis.[68]

Epithelial barrier function, conferred by tight junctions between epithelia, is influenced by nitric oxide. Inhibition of nitric oxide synthesis compromises epithelial barrier dysfunction under basal conditions and in ischaemia reperfusion injury.[52,53,69] Following an acute injury where the epithelial barrier becomes leaky there is a rapid repair/restitution response. This response is impaired by NOS inhibitors,[70] although a contrasting result in the Caco-2 cell line has been reported.[71] Because of the importance of the epithelial barrier in limiting the translocation on luminal flora and contents any impairment of this barrier would promote the inflammatory response. In the acute (iNOS absent) models NO promotes the integrity of this barrier and therefore is anti-inflammatory. In chronic gut inflammation there is also epithelial barrier dysfunction concomitant with exaggerated nitric oxide release,[13,72,73] but in these states NOS inhibitors are protective.

What could be the explanation for this apparent discrepancy? We propose that these differences are mediated by contrasting mechanisms.

Under acute conditions the beneficial effects are mediated by cGMP, indeed cGMP mimics decreased epithelial permeability. In contrast, in iNOS-dependent chronic inflammation the increase in epithelial permeability is the result of increased epithelial cell injury and/or death. It does not matter how tight the tight junctions are if there is rampant epithelial cell death, leukocyte emigration and substantial morphological derangements—crypt abscesses, ulceration and bursting of villus tips. These morphological derangements, characteristic of mucosal inflammation, will promote epithelial barrier dysfunction.

### Pathways of NO metabolism—is injury due to NO or $NO_x$?

Nitric oxide itself is a poorly reactive free radical, and in order for it to be a major component of cellular injury it is likely to be metabolized to alternative species. In an analogous manner superoxide ($O_2^-$) is a weakly reactive reactive oxygen species but it is metabolized in inflammation to a cascade of more reactive products, e.g. HOC1 and $OH^•$. Nitric oxide is inactivated by oxidation to nitrite ($NO_2^-$) and subsequently nitrate ($NO_3^-$) but other oxidized forms may be formed which are not innocuous. Some products require two molecules of nitric oxide as substrate for the generation of the product, e.g. $N_2O_3$ and $N_2O_4$. In other words, these reactions display second-order kinetics and large amounts of the substrate are required to drive the reaction. Thus, it is likely that only in conditions in which iNOS is expressed will these products be formed. One of the consequences of these reactive nitrogen species results from their ability to act as nitrosating and nitrating species. If the target is DNA then this will lead to deamination and point mutations, as indicated in Table 3.[74,75]

*Interaction between $O_2^-$ and NO:* Recently biochemical studies have demonstrated that $O_2^-$ and NO rapidly interact via a radical–radical reaction at a diffusion-limited rate ($k = 6.7 \times 10^9\ M^{-1}.s^{-1}$) to generate the potent oxidant peroxynitrite ($ONOO^-$).[76] Beckman and co-workers[77,78] have suggested that the interaction between these two free radicals to yield $ONOO^-$ and its conjugate acid, peroxynitrous acid (ONOOH), enhances dramatically the toxicity of either $O_2^-$ or NO alone. Indeed, it has been demonstrated *in vitro* using pre-formed or chemically synthesized $ONOO^-$/ONOOH that these oxidants are capable of directly oxidizing carbohydrates,[77] sulfhydryls,[78,79] lipids[80] and DNA bases,[81] as well as mediating bactericidal and endothelial cell toxicity.[77,82,83] It also has been demonstrated that the stimultaneous production of NO and $O_2^-$ by macrophages may result in the formation of $ONOO^-$/ONOOH.[84]

Although $ONOO^-$ is relatively stable, especially at alkaline pH, it has a $pK_a$ of 6.6 which dictates that substantial amounts of this compound will be protonated at physiological pH to yield peroxynitrous acid (ONOOH). This compound is very unstable and has been suggested to rapidly decompose to yield a hydroxyl radical ($OH^•$) and $NO_2^•$-like compound.

$$ONOOH \longrightarrow \text{“}NO_2^• + OH^•\text{”}$$

This hydroxyl radical-like oxidant will react with virtually all biomolecules at diffusion limited rates of reactions ($\sim 10^7$–$10^{10}\ M^{-1}.\ s^{-1}$). Nitrogen dioxide is also a very reactive radical with an ability to react with alkanes and alkenes via free radical-mediated mechanisms.[80,81] Furthermore, Pryor and coworkers have demonstrated that $NO_2^•$ will initiate lipid peroxidation *in vitro*.[85,86] They also demonstrated that abstraction of $H^•$ atoms from unsaturated lipid by $NO_2^•$ resulted in the formation of nitrous acid HONO which was shown to *N*-nitrosate certain amines to yield nitrosamines.[85,86] In addition, $NO_2^•$ is known to react with haemoproteins such as haemoglobin and oxidize thiols and thioethers (methionine).[85,86] Taken together, these data suggest that if the decomposition of ONOOH proceeds via the formation of $OH^•$-like species and $NO_2^•$ then it represents a potentially important pathway in which toxic oxygen and nitrogen-derived free radicals may be formed from the interaction between two relatively unreactive radicals, $O_2^-$ and NO. In fact, this mechanism has been proposed to account for the $O_2^-$ dependent microvascular injury produced by ischaemia and reperfusion of various organ systems.

Because of the ephemeral nature of peroxynitrite it is difficult to measure it *in vitro*, but it can be tracked *in situ* by its ability to promote nitration reactions, in particular the formation of nitrotyrosine. We have demonstrated that nitrotyrosine formation is negligible under basal conditions but in inflammation it is readily demonstrable by immunohistochemistry. Importantly, nitrotyrosine co-localizes with the expres-

**Table 3.** Types of alterations and mutations that may arise from the deamination of DNA bases

| Conversion | | Type of mutation |
|---|---|---|
| Cytosine + $NO_x$ ⟶ | Uracil | G:C ⟶ A:T |
| mCytosine + $NO_x$ ⟶ | Thymine | G:C ⟶ A:T |
| Guanine + $NO_x$ ⟶ | Xanthine | G:C ⟶ A:T |
| Adenine + $NO_x$ ⟶ | Hypoxanthine | A:T ⟶ G:C |

sion of iNOS, and therapy with inhibitors of NOS prevents the formation of nitrotyrosine (and prevents the inflammation). Thus, although nitric oxide itself cannot form nitrotyrosine (it would form nitrosylate not nitrate tyrosine residues), the formation of NO from iNOS is linked to nitrotyrosine formation.

Nitrotyrosine has been demonstrated in several human-disease states—atherosclerosis and amytrophic lateral sclerosis.[87–89] Using HPLC techniques nitrotyrosine was readily detectable in joints of patients with rheumatoid or osteoarthritis[90] and absent in healthy individuals. Furthermore, we have found that nitrotyrosine co-localizes with iNOS and DNA fragmentation in *Helicobacter pylori* gastritis and that eradication of the infection or treatment with the antioxidants, ascorbic acid and/or beta-carotene, reduces the staining for nitrotyrosine.[61]

Studies from Radi's laboratory have clearly demonstrated some of the potential toxic effects of peroxynitrite, including inhibition of cell proliferation, DNA synthesis, succinate dehydrogenase, fumarate reductase, thereby suppressing mitochondrial electron transport chain in the parasite *Trypanosoma*.[91,92] In addition, inhibition of alpha$_1$-proteinase inhibitor has been reported.[93] Direct luminal administration of peroxynitrite can cause colitis in rats.[94]

While it is unlikely that all the deleterious effects of nitric oxide are due to peroxynitrite, the kinetics for the formation of peroxynitrite exceed that for dismutation of superoxide by superoxide dismutase.[77,78] In other words, coproduction of superoxide and nitric oxide will lead to the formation of peroxynitrite unless the NO is sequestered or oxidized. Thus the discrepancies which outline the current debate, i.e. is nitric oxide inherantly anti- or proinflammatory may be moot because the benefit/injury may be mediated by entirely different chemical species. Low doses of nitric oxide are anti-inflammatory whereas high concentrations of NO in conjunction with the release of other reactive oxygen species, will lead to the formation of NO-derived reactive nitrogen species that are pro-inflammatory.

Further evidence that this may be an important explanation is found in pregnancy. Our postulate is that nitric oxide production only has pro-inflammatory effects when it is produced in excess, i.e. when iNOS is expressed. If this postulate is valid then exceptions should not be observed. In other words, are there conditions in which iNOS is expressed but not associated with tissue injury? Pregnancy is the exception. Pregnancy is a state in which iNOS is expressed in a sustained manner in the uterus and placenta[95] but yet is accompanied by cellular/tissue growth and immunosuppression. The explanation for this dichotomy may lie in the production of other oxidants. Unlike inflammatory conditions where nitrotyrosine and iNOS co-localize[26,30] nitrotyrosine is not present in the pregnant uterus or placenta. This suggests that interactions between NO and other reactive oxygen species are critical determinants of the cytotoxic effects of nitric oxide. If pregnant rats are challenged with endotoxin, nitrotyrosine formation and cellular injury can be demonstrated in the utero–placental unit (unpublished data, MJSM). As the synthesis of oxygen free radicals is elevated by endotoxin and inflammation in general, interactions with nitric oxide may be a critical determinant of the role reversal of nitric oxide.

### Mutagenesis, DNA damage and apoptosis

As previously mentioned, NO will spontaneously auto-oxidize to yield potent *N*-nitrosating agents such as $N_2O_3$. This nitrosating agent may then interact with secondary amines to yield potentially carcinogenic nitrosamines. It is known that nitrosamines must be activated into mutagenic and carcinogenic species via the action of the cytochrome P450 system. In addition, it has been demonstrated that electrophilic or mutagenic metabolites may be generated from nitrosamines via a cytochrome P-450 independent pathway involving the interaction of nitrosamines with highly reactive oxy radicals, such as $OH^\bullet$, or with ultraviolet light. Both the P450-dependent and independent pathways appear to involve the formation of the α-hydroxynitrosamine intermediate. This unstable intermediate will spontaneously decompose to yield the monoalkylnitrosamine derivative. Through a series of spontaneous rearrangement and decomposition reactions this intermediate will produce the alkylcarbonium cation which is the ultimate carcinogen. This electrophilic agent will rapidly alkylate a variety of different nucleophilic sites in cellular components including protein, DNA and RNA. It has been determined that alkylation of DNA may occur at as many as twelve different sites including the ring nitrogen positions in adenine, guanine, cytosine and thymine, the oxygen atoms associated with the hydroxyl or carbonyl groups of guanine, thymine and cytosine, and the phosphate groups. Experimental data suggests that $O^6$-alkylation of guanine represents one of the most important reactions because there is a better correlation of the extent of $O^6$-alkylation with carcinogenicity and mutagenicity than with any other type of alkylation reaction. Alkylation of guanine for example would alter the base pairing such that during DNA replication thymine and not cytosine may be incorporated into the newly synthesized strand of the nucleic acid. Mutational effects of this type may then activate certain oncogenes, resulting in malignant transformation.

More recent studies have demonstrated that NO may promote mutagenesis via nitrosative deamination of DNA.[96,97] It has been known for many years that NO-derived nitrosating agents (e.g. $N_2O_3$) will nitrosatively deaminate a variety of primary aromatic amines ($AR\text{-}NH_2$) to yield their hydroxy derivatives. The mechanism appears to involve the *N*-nitrosation of $AR\text{-}NH_2$ to yield a nitrosamine (Ar-NH-NO) intermediate. This unstable intermediate will rapidly rearrange to produce the diazonium ion ($Ar\text{-}N_2^+$) which in turn decomposes to yield the hydroxyl derivative (Ar-OH) of the aromatic amine:

$$Ar\text{-}NH_2 + NO_X \longrightarrow AR\text{-}NH\text{-}NO \longrightarrow ArN_2^+ \longrightarrow AR\text{-}OH$$

From these reactions it has been suggested that deamination of cytosine (C), methyl cytosine (mC), guanine (G) or adenine (A) would result in the formation of uracil (U), thymine (T), xanthine (X), and hypoxanthine (HX), respectively.[97] If these types of reactions occur in intact DNA then base pair substitution mutations may occur. There are two major types of base pair substitution mutations: one type involves the substitution of a purine for purine or a pyrimidine for a pyrimidine and is termed a transition mutation. The other type is called a transversion mutation and is characterized by the substitution of pyrimidine for a purine or vice versa.

Deamination of cytosine to yield uracil for example, will result in the removal of uracil by the enzyme uracil glycosylase leaving an abasic site. This type of lesion is commonly misrepaired by insertion of adenine opposite the site during replication resulting in the observed G:C to A:T base pair substitution mutation (Table 3).[97] If uracil is not repaired it will pair with an incoming adenine to give the same mutation upon replication. Deamination of methyl cytosine to yield thymidine would also produce a G:C to A:T transition (Table 3). This type of mutation is especially significant in mammalian cells because the pattern of methylation of DNA is very important in regulating gene expression and differentiation. In addition to the misrepair of deaminated bases, the instability of hypoxanthine and xanthine in DNA formed from the deamination of adenine and guanine, respectively, leads to rapid depurination forming apurinic sites. It is thought that depurination leads to DNA breakage and subsequent cytotoxicity as well as mutagenicity. Base pair substitution mutations arising from the nitrosative deamination of adenine and guanine would lead to A:T to G:C and G:C to T:A transversion mutations, respectively (Table 3).[97] Based upon the relative rates of NO-dependent deamination of the different bases in DNA, one would expect the G:C to A:T transition mutations to predominate. Indeed, this type of mutation has been shown to predominate in mutants (revertants) of *Salmonella typhimurium* produced by exposure of these cells to NO.[96]

Nitric oxide has been shown to promote apoptosis in cancer cell lines, macrophages and colonic epithelial cells.[68,98,99] The mechanisms by which NO may initiate cell death (apoptosis or necrosis) *vs* uncontrolled cell replication (cancer) may not be as divergent as they superficially appear. Both involve DNA damage. A normal cell whose DNA is damaged by NO, oxidants or the combination, will arrest its cell cycle, undergo DNA repair, or alternatively undergo apoptosis, if the damage is perceived as too extensive. Both the repair and apoptosis processes protect the host from the duplication of damaged DNA, with injured cells repaired or eliminated. Survival of transformed cells may occur if either the repair of apoptosis mechanisms are not effectively evoked. It is commonly considered that cancer results in failures of multiple fail-safes (tumour suppressor and proto-oncogene) and NO may play a role at all levels. In those 'acute' protocols where no deleterious effects are noted, and where high doses of nitric oxide are administered, or in conditions favourable for peroxynitrite formation, an evaluation of DNA status is invariably absent. Unless mutagenesis or induction of apoptosis has been ruled out, there is no assurance that serious complications have not been set into motion. Complications that may remain cloaked to standard physiology-based tests and functional assays.

### Concluding remarks

There is ample evidence that nitric oxide is a molecular aggressor in inflammation. The reported anti-inflammatory actions are either very acute in duration and/or pertain to conditions which are not classically defined as inflammation, but rather states which are primarily driven by oxygen free radicals, e.g. ischaemia/reperfusion injury. Furthermore, NO formation is greatly elevated in inflammation suggesting that if its primary role was anti-inflammatory its actions are too weak to negate tissue damage. Rather, it appears that NO is primarily pro-inflammatory in inflammatory disease states. For this reason new therapeutic approaches for the treatment of chronic inflammatory diseases such as arthritis and inflammatory bowel disease, focus on the inhibition of inducible nitric oxide synthase, and not the use of NO donors.

In the last few years we have come to appreciate that the pro-inflammatory actions of nitric oxide involve a multitude of mechanisms. It is likely that the critical events may involve reactive nitrogen species derived from nitric oxide, but the parent radical is not, in itself, the mediator of tissue destruction. Peroxynitrite, at this time, appears to be the best candidate but other reactive nitrogen species may assume the mantle currently bestowed on peroxynitrite. Further, nitric oxide and related products appear to be chemical links between inflammation and cancer by virtue of their ability to react with DNA bases, causing point mutations.

In summary, nitric oxide is clearly a critical pro-inflammatory mediator. The dilemma established by its anti-inflammatory actions under basal conditions can be reconciled by evaluating the influence of time on the observations, the enzyme source of nitric oxide production, the cellular sources of NO in the condition being evaluated, and the generation of other reactive nitrogen species from NO, particularly those involved in nitrosation and nitration reactions. When these influences are accounted for one can predict if nitric oxide will play an anti- or pro-inflammatory role.

### References

1. Marletta MA. Mammalian synthesis of nitrite, nitrate and *N*-nitrosating agents. *Chem Res Toxicol* 1988; **1:** 249–257.
2. Ignarro LJ, Fukoto JM, Griscavage JM, Rogers NE, Byrns RE. Oxidation of nitric oxide in aqueous solution to nitrite but not nitrate: comparison with enzymatically formed nitric oxide from L-arginine. *Proc Natl Acad Sci USA* 1993; **90:** 8103–8107.
3. Wink DA, Darbyshire JF, Nims RW, Saavedra JE, Ford PC. Reactions of the bioregulatory agent nitric oxide in oxygenated aqueous media: determination of the kinetics for oxidation and nitrosation by intermediates generated in the $NO/O_2$ reaction. *Chem Res Toxicol* 1993; **6:** 23–27.
4. Lewis RS, Deen WM. Kinetics of the reaction of nitric oxide with oxygen in aqueous solutions. *Chem Res Toxicol* 1994; **7:** 568–574.
5. Doyle MP, Hoekstra JW. Oxidation of nitrogen oxides by bound dioxygen in hemoproteins. *J Inorg Biochem* 1981; **14:** 351–358.
6. Drapier JC, Hibbs JB. Differentiation of murine macrophages to express nonspecific cytotoxicity in L-arginine-dependent inhibition of mitochondrial iron-sulphur enzymes in the macrophage effector cells. *J Immunol* 1988; **140:** 2829–2838.
7. Granger DL, Lehninger AL. Sites of inhibition of mitochondrial electron transport in macrophage-induced neoplastic cells. *J Cell Biol* 1992; **95:** 527–535.
8. Drapier JC, Hibbs JB. Murine cytotoxic activated macrophages inhibit aconitase in tumor cells. *J Clin Invest* 1986; **78:** 790–797.
9. Hibbs JB, Tanitor RR, Vavrin Z. Iron depletion: possible cause of tumor cell cytotoxicity induced by activated macrophages. *Biochem Biophys Res Commun* 1984; **123:** 716–723.
10. Boughton-Smith NK, Evans SM, Luzlo F, Whittle BJR, Moncada S. The induction of nitric oxide synthase and intestinal vascular permeability by endotoxin in the rat. *Br J Pharmacol* 1993; **110:** 1189–1195.
11. Lazlo F, Whittle BJR, Moncada S. Time-dependent enhancement or inhibition of endotoxin-induced vascular injury in rat intestine by nitric oxide synthase inhibitors. *Br J Pharmacol* 1994; **11:** 1309–1315.
12. Dudek RR, Wildhirt S, Conforto A, Pinto V, Suzuki H, Winder S, Bing RJ. Inducible nitric oxide synthase activity in myocardium after myocardial infarction in rabbit. *Biochem Biophys Res Commun* 1994; **205:** 1671–1680.
13. Miller MJS, Sadowska-Krowicka H, Chotinaruemol S, Kakkis JL, Clark DA. Amelioration of chronic ileitis by nitric oxide synthase inhibition. *J Pharmacol Exptl Ther* 1993; **264:** 11–16.
14. McCartney-Francis N, Allen JB, Mizel DE, Albina JE, Xie Q-W, Nathan CF, Wahl SM. Suppression of arthritis by an inhibitor of nitric oxide synthase. *J Exptl Med* 1993; **178:** 749–754.
15. Grisham MG, Specian RD, Zimmerman TE. Effects of chronic nitric oxide synthase inhibition on the pathophysiology observed in a model of chronic granulomatous colitis. *J Pharmacol Exp Ther* 1994; **271:** 1114–1121.
16. Connor JR, Manning PT, Settle SL, *et al.* Suppression of adjuvant-induced arthritis by selective inhibitor of inducible nitric oxide synthase. *Eur J Pharmacol* 1995; **273:** 15–24.
17. Wallace JL, Reuter B, Cicala C, McKnight W, Grisham MB, Cirino G. Novel nonsteroidal antiinflammatory drug derivatives with markedly reduced ulcerogenic properties in the rat. *Gastroenterology* 1994; **107:** 173–179.
18. Elliott SN, McKnight W, Cirino G, Wallace JL. A nitric oxide-releasing nonsteroidal antiinflammatory drug accelerates gastric ulcer healing in rats. *Gastroenterology* 1995; **109:** 524–530.
19. Cho HJ, Xie Q-W, Calgary J, Mumford RA, Swiderek KM, Lee TD, Nathan C. Calmodulin is a subunit of nitric oxide synthase from macrophages. *J Exp Med* 1992; **176:** 599–604.
20. Rondouin G, Bockaert J, Lerner-Natoli M. L-Nitroarginine, an inhibitor of NO synthase, dramatically worsens limbic epilepsy in rats. *Neuro Report* 1993; **4:** 1187–1190.
21. Hewett SJ, Corbett JA, McDaniel ML, Choi DW. Inhibition of nitric oxide formation does not protect murine cortical cell cultures from *N*-methyl-D-aspartate neurotoxicity. *Brain Res* 1993; **625:** 337–341.
22. Huang PL, Dawson TM, Bredt DS, Snyder SH, Fishman MC. Targeted disruption of the neuronal nitric oxide synthase gene. *Cell* 1993; **75:** 1273–1286.
23. Lipton SA, Choi Y-B, Pam Z-H, *et al.* A redox-based mechanism for the neuroprotective and nuerodestructive effects of nitric oxide and related nitroso-compounds. *Nature (Lond)* 1993; **364:** 626–632.
24. Iadecola C, Pelligrino DA, Moskowitz MA, Lassen NA. State of the art review: nitric oxide synthase inhibition and cerebrovascular regulation. *J Cereb Blood Flow Metab* 1994; **14:** 175–192.
25. Minc-Golomb D, Tsarfaty I, Schwartz JP. Expression of inducible nitric oxide synthase by neurons following exposure to endotoxin and cytokine. *Br J Pharmacol* 1994; **112:** 720–722.
26. Seago ND, Thompson JH, Zhnag X-J, *et al.* Inducible nitric oxide synthase and guinea-pig ileitis induced by adjuvant. *Mediators of Inflammation* 1995; **4:** 19–24.
27. Nomura Y, Kitamura Y. Inducible nitric oxide synthase in glial cells. *Neuroscience Res* 1993; **18:** 103–107.
28. Iadecola C, Zhang F, Xu X. Inhibition of inducible nitric oxide synthase ameliorates cerebral ischemic damage. *Am J Physiol* 1995; **268:** R286–R292.
29. Koedel U, Beinatowicz A, Paul R, Frei K, Fontana A, Pfister H-W. Experimental pneumococcal meningitis: cerebrovascular alterations, brain edema, and meningeal inflammation are linked to the production of nitric oxide. *Ann Neurol* 1995; **37:** 313–323.
30. Miller MJS, Thompson JH, Zhang X-J, *et al.* Role of inducible nitric oxide synthase expression and peroxynitrite formation in guinea pig ileitis. *Gastroenterology* 1995; **109:** 1475–1483.
31. Aiko S, Grisham MB. Spontaneous intestinal inflammation and nitric oxide metabolism in HLA-B27 transgenic rats. *Gastroenterology* 1995; **109:** 142–150.
32. Stefanovic-Racic M, Stadler J, Evans CH. Nitric oxide and arthritis. *Arthritis Rheumatol* 1993; **36:** 1036–1044.
33. Stefanovic-Racic M, Meyers K, Mescheter C, Coffey JW, Hoffman RA, Evans CH. *N*-monomethyl arginine, an inhibitor of nitric oxide synthase, suppresses the development of adjuvant arthritis in rats. *Arthritis Rheum* 1994; **37:** 1062–1069.
34. Järvinen TAH, Moilanen T, Järvinen TLN, Moilanen E. Nitric oxide mediates interleukin-1 induced inhibition of glycocaminoglycan synthesis in rat articular cartilage. *Mediators of Inflammation* 1995; **4:** 107–111.
35. Ialenti A, Moncada S, Di Rosa M. Modulation of adjuvant arthritis by endogenous nitric oxide. *Br J Pharmacol* 1993; **110:** 701–706.
36. Weinberg JB, Granger DL, Pisetsky DS, *et al.* The role of nitric oxide in the pathogenesis of spontaneous murine autoimmune disease: increased nitric oxide production and nitric oxide synthase expression in MRI-1pr/1pr mice, and reduction of spontaneous glomerulonephritis and arthritis by orally administered $N^G$-monomethyl-L-arginine. *J Exp Med* 1994; **179:** 651–659.
37. Cross AH, Misko TP, Liu RF, Hickey WF, Trotter JL, Tilton RG. Aminoguanidine, an inhibitor of inducible nitric oxide synthase, ameliorates experimental autoimmune encephalomyelitis in SJL mice. *J Clin Invest* 1994; **93:** 2684–2690.
38. Miller MJS, Munshi UK, Sadowska-Krowicka H, Kakkis JL, Zhang X-J, Eloby-Childress S, Clark DA. Inhibition of calcium-dependent nitric oxide synthase causes ileitis and leukocytosis in guinea pigs. *Dig Dis Sci* 1994; **39:** 1185–1192.
39. Miller MJS, Clark DA. Nitric oxide inhibition can initiate or prevent gut inflammation: role of enzyme source. *Agents and Actions* 1994; **41:** C231–C232.
40. Miller MJS, Munshi UK, Zhang X-J, *et al.* Chronic administration of the nitric oxide synthase inhibitor, L-NAME, increases circulating endothelin levels in guinea pigs. *Endothelium* 1994; **3:** 57–62.
41. Worrall NK, Lazentry WD, Misko TP, *et al.* Modulation of *in vivo* alloreactivity by inhibition of inducible nitric oxide synthase. *J Exp Med* 1995; **181:** 63–70.
42. Rachmilewitz D, Karmeli F, Okon E. Sulfhydryl blocker-induced rat colonic inflammation is ameliorated by inhibition of nitric oxide synthase. *Gastroenterology* 1995; **109:** 98–106.
43. Lindsay RM, Smith W, Rossiter SP, McIntyre MA, Williams BC, Baird JD. $N^{\omega}$-nitro-L-arginine methyl ester reduces the incidence of IDDM in BB/E rats. *Diabetes* 1995; **44:** 365–368.
44. Tracey WR, Nakame M, Kulk J, Budzik G, Klinghoffer V, Harris R, Carter G. The nitric oxide synthase inhibitor, L-$N^{G}$-monomethylarginine, reduces carrageenin-induced pleurisy in the rat. *J Pharmacol Exptl Ther* 1995; **273:** 1295–1299.
45. Ianaro A, O'Donnell CA, Di Rosa M, Liew FY. A nitric oxide synthase inhibitor reduces inflammation, down-regulates inflammatory cytokines and enhances interleukin-10 production in carrageenin-induced edema in mice. *Immunology* 1994; **82:** 370–375.
46. Parks DJ, Cheung MK, Chan G-C, Roberge FG. The role of nitric oxide in uveitis. *Arch Opthalmol* 1994; **112:** 544–546.
47. Vane JR, Mitchell JA, Appleton I, Tomlinson A, Bishop-Bailey D, Croxtall J, Willoughby DA. Inducible isoforms of cyclooxygenase and nitric oxide synthase in inflammation. *Proc Natl Acad Sci USA* 1994; **91:** 2046–2050.
48. Hogaboam CM, Jacobson K, Collins SM, Blennerhassett MG. The selective beneficial effects of nitric oxide inhibition in experimental colitis. *Am J Physiol* 1995; **268:** G673–G684.
49. Rachmilewitz D, Karmeli F, Okon E, Bursztyn M. Experimental colitis is ameliorated by inhibition of nitric oxide synthase activity. *Gut* 1995; **37:** 247–255.
50. Ribbons KA, Zhang X-J, Thompson JH, *et al.* Potential role of nitric oxide in a model of chronic colitis in rhesus macaques. *Gastroenterology* 1995; **108:** 705–711.
51. Kubes P. Nitric oxide-induced microvascular permeability alterations: a regulatory role for cGMP. *Am J Physiol* 1993; **265:** H1909–H1915.
52. Payne D, Kubes P. Nitric oxide donors reduce the rise in reperfusion-induced intestinal mucosal permeability. *Am J Physiol* 1993; **265:** G189–G195.
53. Kanwar S, Wallace JL, Befus D, Kubes P. Nitric oxide synthesis inhibition increases epithelial permeability via mast cells. *Am J Physiol* 1994; **266:** G222–G229.
54. Ma T, Ischiropoulos H, Brass CA. Endotoxin-stimulated nitric oxide production increase injury and reduces rat liver chemiluminescence during reperfusion. *Gastroenterology* 1995; **108:** 463–469.
55. Nava E, Palmer RMJ, Moncada S. Inhibition of nitric oxide synthesis in septic shock: how much is beneficial? *Lancet* 1991; **338:** 1555–1557.
56. Greenberg SS, Xie J, Zatarain JM, Kapusta DR, Miller MJS. Hydroxocobalamin (vitamin B12a) prevents and reverses endotoxin-induced hypotension mortality in rodents: role of nitric oxide. *J Pharmacol Exp Ther* 1995; **273:** 257–265.
57. Middleton SJ, Shorthouse M, Hunter JO. Increased nitric oxide synthesis in ulcerative colitis. *Lancet* 1993; **341:** 465–466.
58. Boughton-Smith NK, Evans SM, Cole AT, Hawkey CJ, Whittle BJR, Moncada S. Induction of nitric oxide synthase in inflamed colon from ulcerative colitis patients. *Lancet* 1993; **341:** 338–340.
59. Roediger WEW, Lawson MJ, Nance SH, Radcliffe BC. Detectable colonic nitrite levels in inflammatory bowel disease—mucosal or bacterial malfunction? *Digestion* 1986; **35:** 199–204.
60. Rachmilewitz D, Karmeli F, Eliakim, Stalnikowicz R, Ackerman Z, Amir G, Stamler JS. Enhanced gastric nitric oxide synthase activity in duodenal ulcer patients. *Gut* 1994; **35:** 1394–1397.
61. Bravo LE, Mannick EE, Zhang X-J, Ruiz B, Correa P, Miller MJS. *H. pylori* infection is associated with inducible nitric oxide synthase expression, nitrotyrosine and DNA damage. *Gastroenterology* 1995; **108:** A63 (abstract).
62. Farrell AJ, Blake DR, Palmer RMJ. Moncada S. Increased concentrations of nitrite in synovial fluid and serum samples suggest increased nitric oxide synthesis in rheumatic diseases. *Ann Rheum Dis* 1992; **51:** 1219–1222.
63. Nakane M, Schmidt HHHW, Pollack JS, Förstermann U, Murall F. Cloned human brain nitric oxide synthase is highly expressed in skeletal muscle. *FEBS Letters* 1993; **316:** 175–180.
64. Lowik CWGM, Nibbering PH, Van de Ruit M, Papapoulos SE. Inducible production of nitric oxide in osteoblast-like cells and in fetal mouse bone explants is associated with suppression of osteoclastic bone resorption. *J Clin Invest* 1994; **93:** 1465–1472.
65. Ralston SH, Todd D, Helfrich M, Benjamin N, Grabowski PS. Human osteoblast-like cells produce nitric oxide and express inducible nitric oxide synthase. *Endocrinology* 1994; **135:** 330–336.
66. Tepperman BL, Brown JF, Whittle BJR. Nitric oxide synthase induction and intestinal epithelial cell viability in rats. *Am J Physiol* 1993; **265:** G214–G218.
67. Tepperman BL, Brown JF, Korolkiewiez, Whittle BJR. Nitric oxide synthase activity, viability and cyclic GMP levels in rat colon epithelial cells: effect of endotoxin challenge. *J Pharmacol Exptl Ther* 1994; **271:** 1477–1482.
68. Sandoval M, Liu X, Oliver PD, Zhang X-J, Clark DA, Miller MJS. Nitric oxide induces apoptosis in a human colonic epithelial cell line, T84. *Mediators of Inflammation* 1995; **4:** 248–250.
69. Kubes P. Ischemia-reperfusion in feline small intestine: a role for nitric oxide. *Am J Physiol* 1993; **265:** G143–G149.
70. Miller MJS, Zhang X-J, Sadowska-Krowicka H, Chotinaruemol S, McIntyre JA, Clark DA, Bustamante SA. Nitric oxide release in response to gut injury. *Scand J Gastroenterol* 1993; **28:** 149–154.
71. Salzman AL, Menconi MJ, Unno N, Ezzell RM, Casey DM, Gonzalez PK, Fink MP. Nitric oxide dilates tight junctions and depletes ATP in cultured Caco-2BBe intestinal epithelial monolayers. *Am J Physiol* 1995; **268:** G361–G373.
72. Yamada T, Sartor RB, Marshall S, Specian RD, Girsham MB. Mucosal injury and inflammation in a model of chronic granulomatous colitis in rats. *Gastroenterology* 1993; **104:** 759–771.
73. Miller MJS, Zhang X-J, Barkemeyer B, Grisham MB, Sadowska-Krowicka H, Eloby-Childress S, Clark DA. Rabbit gut permeability in response to histamine chloramines and chemotactic peptide. *Gastroenterology* 1992; **103:** 1537–1546.
74. Bartsch HE, Hietanen E, Molaveille C. Carcinogen nitrosamines: free radical aspects of their action. *Free Radical Biol Med* 1989; **7:** 637–644.
75. Grisham MB, Ware K, Gilleland HE Jr, Gilleland LB, Abell CL, Yamada T. Neutrophil-mediated nitrosamine formation: role of nitric oxide in rats. *Gastroenterology* 1992; **103:** 1260–1266.
76. Huie RE, Padmaja S. The reaction of NO with superoxide. *Free Rad Res Commun* 1993; **18:** 195–199.
77. Beckman JS, Beckman TW, Chen J, Marshall PA, Freeman BA. Apparent hydroxyl radical production by peroxynitrite: implications for endothelial injury from nitric oxide and superoxide. *Proc Natl Acad Sci (USA)* 1990; **87:** 1620–1624.
78. Radi R, Beckman JS, Bush KM, Freeman BA. Peroxynitrite oxidation of sulhydryls. *J Biol Chem* 1991; **266:** 4244–4250.
79. Rubbo H, Denicola A, Radi R. Peroxynitrite inactivates thiol-containing enzymes of *Trypanosoma cruzi* energetic metabolism and inhibits cell respiration. *Arch Biochem Biophys* 1994; **308:** 96–102.
80. Radi R, Beckman JS, Bush KM, Freeman BA. Peroxynitrite-induced membrane lipid peroxidation. The cytotoxic potential of superoxide and nitric oxide. *Arch Biochem Biophys* 1991; **288:** 481–487.
81. King PA, Jamison E, Strahs D, Anderson VE, Brenowitz M. 'Footprinting' proteins on DNA with peroxonitrous acid. *Nucleic Acids Research* 1993; **21:** 2473–2478.
82. Zhu L, Gunn C, Beckman JS. Bactericidal activity of peroxynitrite. *Arch Biochem Biophys* 1992; **298:** 452–457.
83. Kooy NW, Royall JA. Agonist-induced peroxynitrite production from endothelial cells. *Arch Biochem Biophys* 1994; **310:** 352–359.
84. Ischiropoulos H, Zhu L, Beckman JS. Peroxynitrite formation from macrophage-derived nitric oxide. *Arch Biochem Biophys* 1992; **288:** 446–451.
85. Pryor WA, Lightsey JW. Mechanisms of nitrogen dioxide reactions: Initiation of lipid peroxidation and the production of nitrous acid. *Science* 1981; **214:** 435–437.
86. Pryor WA. Oxy-radical and relates species: their formation lifetime and reactions. *Ann Rev Physiol* 1986; **48:** 657–667.
87. Ischiropoulous H, Zhu L, Chen J, Tsai M, Martin JC, Smith CD, Beckman JS. Peroxynitrite-mediated tyrosine nitration catalyzed by superoxide dismutase. *Arch Biochem Biophys* 1992; **298:** 431–437.
88. Beckman JS, Ye YZ, Anderson PG, Chen J, Accavitti MA, Tarpey MM, White CR. Extensive nitration of protein tyrosines in human atherosclerosis detected by immunohistochemistry. *Biol Chem* Hoppe-Seyler 1994; **375:** 81–88.
89. Beckman JS, Crow JP. Pathophysiological implications of nitric oxide, superoxide and peroxynitrite formation. *Biochem Soc Transactions* 1993; **21:** 330–334.
90. Kaur H, Halliwell B. Evidence of nitric oxide-mediated oxidative damage in chronic inflammation. Nitrotyrosine in serum and synovial fluid from rheumatoid patients. *FEBS Lett* 1994; **350:** 9–12.
91. Denicola A, Rubbs H, Rodriguez D, Radi R. Peroxynitrite-mediated cytotoxicity to *Trypanosoma cruzi*. *Arch Biochem Biophys* 1993; **304:** 279–286.
92. Radi R, Rodriguez M, Castro L, Telleri R. Inhibition of mitochondrial electron transport by peroxynitrite. *Arch Biochem Biophys.* 1994; **308:** 89–95.
93. Moreno JJ, Pryor WA. Inactivation of α1-proteinase inhibitor of peroxynitrite. *Chem Res Toxicol* 1992; **5:** 425–431.
94. Rachmilewitz D, Stamler JS, Karmeli F, *et al.* Peroxynitrite-induced rat colitis—a new model of colonic inflammation. *Gastroenterology* 1993; **105:** 1681–1686.
95. Natuzzi ES, Ursell PC, Harrison M, Buschner C, Riemer RK. Nitric oxide synthase activity in the pregnant uterus decreases at parturition. *Biochem Biophys Res Commun* 1993; **194:** 1–8.
96. Wink DA, Kasprzak KS, Maragos CM, *et al.* DNA deaminating ability and genotoxicity of nitric oxide and its progenitors. *Science* 1991; **254:** 1001.
97. Nguyen T, Brunson D, Crespit CL, Penman BW, Wishnok JS, Tannenbaum SR. DNA damage and mutation in human cells exposed to nitric oxide *in vitro*. *Proc Natl Acad Sci USA* 1992; **89:** 3033–3034.
98. Albina JE, Cui S, Mateo RB, Reicher JS. Nitric oxide-mediated apoptosis in murine peritoneal macrophages. *J Immunol* 1993; **150:** 5080–5085.
99. Cui S, Reichner JS, Mateo RB, Albina JE. Activated murine macrophages induces apoptosis in tumor cells through nitric oxide-dependent or-independent mechanisms. *Cancer Res* 1994; **54:** 2462–2467.

ACKNOWLEDGEMENTS. Present and past funding from the NIH and Crohn's & Colitis Foundation of America have contributed to the authors' research in this field.

**Received 11 September 1995; accepted 13 September 1995**
